# Supplementary material for: Developing a context-relevant psychosocial stimulation intervention to promote cognitive development of children with severe acute malnutrition in Mwanza, Tanzania
Source: PLoS One. 2024 May 9;19(5):e0285240. doi: 10.1371/journal.pone.0285240 (PMC11081340; doi:10.1371/journal.pone.0285240)
Supplement: Attachment — Submitted filename: Response to reviewers.docx [file pone.0285240.s002.docx]

**Response to Reviewers**

Below we provide a point-by-point response to the comments made by reviewers. We have revised our manuscript based on comments and suggestions and would like to acknowledge that these inputs have improved our manuscript. The line numbers refer til the clean version of the revised manuscript (file labeled “Manuscript“).

Reviewers' comments:

**Reviewer #1:**

The topic of this study is highly significant at both local and global levels. Investment in promoting early childhood development can yield substantial health, social, and economic benefits for society as a whole. There is a dearth of psychosocial stimulation programs designed for children facing adversities in resource-constrained settings. However, the study could have several flaws, leading to limited results. No results on the barriers and facilitators at the child, community, healthcare providers, and the healthcare system levels were reported.

Below are my suggestions:

1. Avoid excessive use of acronyms.

Response: In the revised manuscript, we have reduced the use of acronyms.

2. Introduction: Provide a comprehensive explanation of psychosocial stimulation, both in general and specific to Severe Acute Malnutrition (SAM). Detail the WHO guidelines for inpatient SAM treatment and recommendations for psychosocial stimulation.

Response: We acknowledge that information was lacking on these topics and have thus made major revisions to the introduction section to include details about severe acute malnutrition, psychosocial stimulation and the relevance of stimulation interventions for children after exposure to SAM (line 59-107).

3. Methods: Provide a more detailed description of the design of the qualitative formative research in the "Study Design and Setting" section. Explain the rationale behind the chosen qualitative methods and the theoretical framework guiding the study.

Response: We have added a detailed description to the suggested section of the revised version (line 125-147).

4. Clarify whether the psychosocial interventions studied were intended for inpatient treatment in hospitals or community management of SAM.

Response: Our intention was to develop a psychosocial intervention that can be integrated with management of SAM both when delivered in hospitals and in communities. We have clarified this in the revised version (line 118-119).

5. Describe the process of selecting professionals for the study.

Response: The professionals (pediatrician, nutritionist, nurses in malnutrition ward, and key staff from TAHEA and EFFECTS) were also purposively selected based on their experience in working/ involvement with SAM children or projects related to malnutrition and/or early child development. We have added a description to the revised manuscript (line 169-173)

6. Specify the number of participants who declined to participate and their reasons for refusal.

Response: We have added these details to the first paragraph of the results section (line 250-253).

7. Elaborate on the process of deciding to stop conducting interviews based on data saturation. Explain who made this decision and how.

Response: The senior social scientist (E.S.) made this decision based on ongoing preliminary analysis of the interviews. We have added a more detailed description to the revised manuscript (line 193-197).

8. Detail the development and pilot testing of the interview guides.

Response: We have revised and re-organised the methods section to improve the description of the development and testing of the interview guides, which was previously spread over several paragraphs. The revised version now includes more detail on these processes (line 175-187).

9. Address the limitations of the study's results, particularly their focus on barriers and facilitators at the family level.

Response: Our study focused mainly on barriers and facilitators for supporting early child development at the family level, as the study was intended to inform development of an intervention delivered to families while in contact with health care teams during management of severe acute malnutrition. In addition, our topic guide focused on the views of caregivers and thus may have been biased to look more within the family. Although the focus of our results was on the family situation, our results do include barriers and facilitators at other levels as these emerged inductively when we coded the data, e.g. relating to ‘illness’ and ‘malnutrition’ at the child level, and we mention the need for links between nutrition units at the healthcare system level and community workers. In the revised manuscript, we now explain this as part of the methods section (line 178-182) and we’ve included it as a discussion point in the study limitations (line 526-536)

**Reviewer #2:**

This is a timely and insightful research related to child development and malnutrition particularly in low and middle income countries. It unearths key findings that are useful for interventions in similar context. It also provides nuanced information given the paucity of qualitative studies on the subject matter. While this is a well written manuscript, some aspects of the paper require revision.

The introductory information that builds up argument for the study appear limited. For instance, what is severe acute malnutrition (SAM)?, What does psychosocial stimulation entail and/or what are its components?. The introduction section can improve with a bit more information to better ground the study.

Response: We agree that the introduction could be improved. This was also commented by reviewer 1 and we have therefore rewritten the introduction section to improve the explanations of SAM, psychosocial stimulation and the relevance of providing stimulation to children exposed to SAM (line 59-107)

The study was guided by the nurturing care framework (NCF) by the WHO. Data was organized and synthesized using this framework. However, the framework has only been summarized without being thoroughly reviewed. For instance, what are its strengths or otherwise?, why was it the best option for this study and not some other framework out there?

Then again, what do the five components of the NCF mean. When you refer to ‘good health’ or ‘adequate nutrition’ etc , what does this mean for your study. In effect, if the framework was thoroughly reviewed, it will clear all doubts and help one see if findings reflect what you set out to do.

Response: We have provided more details about the NCF and our motivation for choosing this framework in the revised version (line 140-147).

You state how caregivers were sampled (purposively). How were professionals sampled? How did you decide which professional to interview or were all professionals within the context of interest interviewed?

Response: We acknowledge that this information was missing from the previous version, as also noticed by reviewer 1, and have now added the details to the method section (line 169-173).

Under the results section, on page 10 you provide a quote from a professional on the wellbeing of the caretaker. Linking this to the comment above about how dearth the explanation of the NCF framework is, it becomes unclear exactly who the focus of the component of ‘good health’ is for. If the components are well explained, then it will be easier to link or see the barriers to promoting PS for children in SAM.

Response: The comment relates to a quote about the importance of considering the health of caregivers as their (mental) health is central for them being able to take on messages about ECD and provide responsive care for their children. We agree with the reviewer that it could be argued that this does not belong with the “health” component of NCF which is mainly about the child’s health. In our revision, we have moved this part to the component on “awareness and prioritization of play and opportunities for early learning” as we believe its main message has to do with competing concerns and prioritization among caregivers (line 389-395).

On the component of ‘security and safety’ on page 15, it is stated that there was limited discussion on psychological stress. Can a brief reason be given since the study’s focus is on PS?

Response: This comment refers to a statement that was intended to say that there was limited discussion on the physical aspects of ECD (safety and security). We acknowledge that the statement was unclear and could be understood as limited discussion on the psychological stress. We have revised to clarify this (line 408-411). However, we also acknowledge that there may have been more discussion on physical aspects if we had probed specifically into this during interviews. We have added this as a limitation in the discussion section (line 526-536).
