# Supplementary material for: Developing a context-relevant psychosocial stimulation intervention to promote cognitive development of children with severe acute malnutrition in Mwanza, Tanzania
Source: PLoS One. 2024 May 9;19(5):e0285240. doi: 10.1371/journal.pone.0285240 (PMC11081340; doi:10.1371/journal.pone.0285240)
Supplement: Attachment — Submitted filename: Response to reviewers2.docx [file pone.0285240.s003.docx]

**Response to Reviewers’ Comments**

Below we provide a point-by-point response to the second round of comments made by reviewers. We have revised our manuscript further based on these additional comments and suggestions and would like to acknowledge that these inputs have improved our manuscript. The line numbers refer til the clean version of the revised manuscript (file labeled “Manuscript“).

Reviewers' comments:

**Reviewer #2:**

The paper makes a valuable contribution to global health research and provides nuanced insights into using psychosocial stimulations to improve the well being and health outcomes of children in poor resourced countries.

The introduction of the paper has been restructured making it clearer and concise. There is less use of acronyms and this has boosted clarity.

The methods used have now been clearly explained and details the framework (NCF) that was used to analyze the data. Also good to see some reflexivity on the part of authors and research participants, as this allows for transparency.

There is also an insightful engagement with the findings in addition to providing contextual information removing any ambiguities. An outline of limitations has made the paper more focused. Bar one or two grammatical omissions, the paper is robust enough to be published.

Response: We thank the reviewer for following up on the comments previously made. We are glad to learn that the reviewer found our last round of revisions useful, and we note that the reviewer has no further comments.

**Reviewer #3:**

I read the manuscript with keen interest. Overall, it is of relevance and well written. It also followed a scientific structure. Data management and analysis was done appropriately. Verbatim translation of recordings was done and this would ensure originality of contents. The coding process as described was thorough and the application of thematic content analysis was appropriate. A reliable tool (Dedoose) was used for the data processing/analysis. However, the authors’ statement on data availability gives an indication that access to data to supplement the manuscript is highly restrictive. The framework used has to be illustrated clearly to relate well with the results and discussions. The results should be clearly presented to reflect the framework. The discussions should also reflect same. For instance, it was surprising that the authors mentioned “social”, “environmental” and “familial" factors in their discussion. These were not indicated anywhere in the results and methods section where the framework was mentioned.

Response: We thank the reviewer for the positive feedback. We also acknowledge that access to the data from this study is highly restrictive under the current data protection regulations in Tanzania. However, data can be made available to other researchers upon reasonable request. We have given the relevant contact info in our ‘data availability statement’. Lastly, we agree with the reviewer that the Nurturing Care Framework could be further elaborated in relation to our presentation of results and discussion. We describe how we have done this in the point-by-point responses below.

*Study design and setting*

• A brief description of the ‘BrightSAM (Brain development, growth and health in children with SAM) trial development study’ would help. Otherwise, it could be cited that this has been described elsewhere (e.g. reports, protocol, etc.).

Response: A brief description is given in the first paragraph of the Methods section. We have now added a reference to a recent publication which included additional details about the study (Mwita *et al.*, 2024). (Line 113-114)

• Sentence construction in line 135: It should be “exploring the views and experiences of a range "of" caregivers. The second "of" is missing.

Response: Thank you. The typo has now been corrected. (Line 145)

• The framework used (Nurturing Care Framework) should be elaborated further to illustrate how the barriers and facilitators interact with the five components (good health, adequate nutrition, responsive caregiving, opportunities for early learning, and security and safety). Also, I suggest the framework is given a section/sub-section with a clear heading to make it more visible in the manuscript.

Response: We have added a sub-heading to the paragraph describing the theoretical framework used. We aimed to explore facilitators and barriers in promoting early child development among children treated for SAM. We applied the structure of the framework with its 5 components to organize and interpret our findings, as we found that the expressed barriers and facilitators were each linked with one or more of the components. We have clarified this in the revised version (Line 150-160)

• In terms of flow, the description of the study setting as in line 149-160 could be moved to the beginning of the sub-section (study design and setting).

Response: We agree and have restructured the Methods section accordingly (Line 122-133)

*Participant selection and data collection*

• The authors mentioned that “sampling was done with assistance from healthcare staff from the nutrition unit at BMC”. It would be clearer if this is elaborated a little further. How did the health staff help? What role did they play? Did they play a role in recruitment of the participants? If they played a role in recruitment, this could have influenced caregivers’ responses to questions relating to the health facility and caregiver. In that case, using the healthcare staff to recruit participants could introduce response biases which could be a limitation of the study. Such a limitation is worth mentioning in the manuscript.

Response: We agree that involvement of healthcare providers could bias the responses given in a qualitative study about health services. However, in the present study, the health staff only helped the researchers to identify mothers with children who had recently been treated for SAM. They gave us their phone numbers and played no further role in the study. All communication about the study was with our study staff who had not been involved in the treatment of the child. We have clarified this in the revised version. (Line 165-168)

• The number of interviews by category should be indicated. Also, an explanation of how such numbers were arrived at would be useful.

Response: We’ve added the number of interviews by category of professionals to the Methods section (Line 179-182). In the previous version, we explained that the number of interviews was based on the principle of data saturation, but this only applies to the interviews with caregivers. The number of interviews with professionals was based on an ambition to include all categories of staff with relevant experience of treatment of severe acute malnutrition and/or promotion of child development in the setting. This has now been clarified in the revised manuscript (Line 177-179). We’ve also rearranged the text to give the information about numbers of interviews for both groups (caregivers and professionals) in the first paragraphs of ‘Participant selection and data collection’ (Line 164-184).

*Results*

• Is it possible to know how many participants were from the NGO and how many were healthcare workers? This should have also been addressed in the participant selection section.

Response: As described above, we’ve added the number of interviews by category to the method section as this was pre-defined by the availability of professionals with relevant experience. We refer back to these numbers in the result section to avoid repeating the information (Line 256).

• From the way the results are presented, the perspectives on suggestions and recommendations do not clearly standout. They are mixed with issues that came up in the perspectives on barriers and facilitators making it difficult for a reader to follow. Perhaps a separate heading could be created for suggestions and recommendations.

Response: We’ve structured the findings according to the 5 components of the NCF framework and we present these in a subsection called ‘Barriers and facilitators for providing psychosocial stimulation’. We agree with the reviewer that this subsection also presents suggestions and recommendations as these are closely linked with the barriers/facilitators. In line with the reviewer’s suggestion, we have now revised the following subsection called ‘Suggestions and recommendations for psychosocial stimulation in SAM management’ in which we sum up the relevant findings that can inform the design of a context-specific psychosocial stimulation package to be integrated with SAM management (our second aim of the study) (Line 430-442)

• In the summary (as indicated in the framework), it is not clear whether all the recommendations were perspectives of the study participants or they were based on the authors’ objectivity based on their analysis.

Response: This section has now been revised to give the suggestions and recommendations for developing a PS package while more clearly indicating how these are supported by the study’s findings (line 430-442)

*Discussions*

• Line 436-468: the authors said “Our results demonstrate social, environmental and familial factors….”. From the results section, which of the factors are categorized as social, environmental and familial factors? This should be made clear either in the results section or in the discussion.

Response: This was meant as a general characterization of our findings rather than a formal categorization. We have now revised to avoid introducing new terms in the description of findings (Line 446-447).

**Reference**

Mwita, F.C. *et al.* (2024) ‘Developmental and Nutritional Changes in Children with Severe Acute Malnutrition Provided with n-3 Fatty Acids Improved Ready-to-Use Therapeutic Food and Psychosocial Support: A Pilot Study in Tanzania’, *Nutrients*, 16(5), p. 692. Available at: https://doi.org/10.3390/nu16050692.
